# Strain Elastography of Injured Equine Superficial Digital Flexor Tendons: A Reliability Study of Manual Measurements

**DOI:** 10.3390/ani11030795

**Published:** 2021-03-12

**Authors:** Valentina Secchi, Gerolamo Masala, Andrea Corda, Francesca Corda, Enrica Potop, Alicia Barbero Fernandez, Maria Luisa Pinna Parpaglia, Eraldo Sanna Passino

**Affiliations:** 1 Department of Veterinary Medicine, University of Sassari, 07100 Sassari, Italy; valentinasecchi.vet@gmail.com (V.S.); gemasala72@gmail.com (G.M.); francescacorda91@tiscali.it (F.C.); enrica.potop@yahoo.it (E.P.); pinnapar@uniss.it (M.L.P.P.); esp@uniss.it (E.S.P.); 2 Veterinary Teaching Hospital, University of Sassari, 07100 Sassari, Italy; 3 Comparative Surgery Research Laboratory, University of Sassari, 07100 Sassari, Italy; 4 Department of Veterinary Medicine, University Alfonso X el Sabio, 28691 Villanueva de la Cañada, Madrid, Spain; aliciabarbero.vet@gmail.com

**Keywords:** horse, strain elastography, SDFT, manual measurement, ImageJ, reliability

## Abstract

**Simple Summary:**

Strain elastography is an ultrasound-based technique that assesses the mechanical properties of tissues and gives a relative representation of elasticity. Early diagnosis of tendon injuries and long-term monitoring of the healing process are key to equine practice; thus, an accurate method is needed for analyzing and interpreting the images obtained with strain elastography. The first aim of the study was to demonstrate the intraoperator repeatability and interoperator reproducibility of manual measurements of elastograms obtained from injured superficial digital flexor tendons of horses; the second aim was to perform a standardization of the manual measurement method by comparing it with external software. Despite their subjectivity, manual measurements proved to be repeatable and reproducible. In addition, the results obtained with the manual method matched those obtained with the external software.

**Abstract:**

Early diagnosis of tendon injuries and accurate long-term monitoring of the healing process are key for equine veterinarians that use conventional ultrasonography. The development of strain elastography could improve the management of clinical cases. The aim of the study was to assess the intraobserver repeatability and interobserver reproducibility of manual measurements of the colored areas of the tendons within elastograms and to standardize this manual modality by comparing the analysis of the images with ImageJ. Twenty elastograms of the injured superficial digital flexor tendons (SDFTs) of horses were analyzed by two different operators after an acute injury was diagnosed with ultrasonography. Statistical analysis demonstrated excellent intraobserver repeatability (intraclass correlation coefficient, ICC = 0.949) and good interobserver reproducibility (ICC = 0.855) for manual measurements performed with tools available on the ultrasound unit. A good agreement between manual measurements and measurements performed with ImageJ (ICC = 0.849) was then demonstrated. Despite its subjectivity, the manual modality proved to be a valid method for analyzing images obtained with strain elastography.

## 1. Introduction

Sports horses are particularly subject to tendon injuries. This is a huge issue due to the high recurrence rate and the time needed for complete healing [1,2]. Two-dimensional ultrasonography (2D-US) is commonly used to diagnose and monitor equine tendinopathies. In fact, due to the use of portable ultrasound units, 2D-US is low-risk and easy to use in the field. However, it is not able to assess the mechanical properties of tissues, or to predict injuries or detect abnormal signs in tendons over five months post-injury, even though tendons may need up to 18 months for a total recovery [1,3,4].

On the other hand, ultrasound elastography (USE) is able to assess the mechanical properties of tissues and provide information on their elasticity. The two main types of USE are shear wave elastography (SWE) and strain elastography (SE).

Shear wave elastography is a quantitative technique based on the propagation of shear waves, thanks to a force applied on the tissues and created by the ultrasound unit.

Strain elastography, also called real-time elastography or compression elastography, is a qualitative or semi-quantitative USE technique. It is performed by applying a gentle manual rhythmic compression on tissues with the transducer, causing an axial displacement, higher for more elastic (softer) structures and lower for less elastic (harder) ones. Dedicated ultrasound elastography software calculates the axial displacement by comparing 2D-US images pre- and post-compression, thus obtaining a color-coded strain map (elastogram), superimposed on the greyscale image.

Unlike SWE, SE does not produce numerical elasticity parameters and provides a relative representation of the elasticity of tissues within the selected window, field of view (Fov). Colors in the elastogram thus depend on the structures in the Fov, which must be kept constant throughout ultrasound examinations for a correct comparison of images [5].

Despite being highly operator-dependent and its relativity, SE is a feasible, repeatable and reproducible method for assessing normal tendons and ligaments of equine distal limbs [6]. It can also be used to detect early tendinopathies and to evaluate tendon healing, if always associated with 2D-US [7,8,9,10,11].

Over five months post-injury, during the healing process, 2D-US would seem unable to recognize subtle abnormalities in tendon structures [3,12].

In contrast, SE is able to identify small differences in the mechanical properties of the affected area compared to the surrounding healthy tendon. It could thus be useful in evaluating tendon healing, even during the late phase of rehabilitation, because of its higher sensitivity than 2D-US. This would help clinicians to establish a more accurate prognosis for clinical cases [12].

Another important aspect to be considered for the correct clinical application of SE is an accurate evaluation and interpretation of images after the acquisition. A comprehensive and objective analysis of the color patterns identified by SE could be key in assessing the healing process of tendons.

However, due to the lack of objective and numeric parameters, SE elastograms are difficult to interpret, and only a subjective and categorical classification is usually performed [6,11,13,14].

An alternative method for image analysis using data from external software could help to make manual modality more objective.

Although image analysis with external software is more objective, it is also a complex and long procedure, and thus is impracticable in daily clinical activities. On the other hand, taking manual measurements with the ultrasound unit is a simpler method and applicable in the field, but it is also highly operator-dependent and subjective.

This problem could be avoided by standardizing the procedure with external software. Consequently, the first aim of our study was to assess the intraobserver repeatability and interobserver reproducibility of manual measurements of colored areas (red, green and blue) in the elastograms of injured tendons, obtained with an ultrasound unit equipped with SE software.

The second aim was to standardize the manual measurement modality, using external software for image analysis.

## 2. Materials and Methods

### 2.1. Horses and Preparation

Twenty Anglo-Arabian racehorses (11 mares and 9 geldings, aged between 4 and 9 years) with a forelimb superficial digital flexor tendon (SDFT) core lesion, which had occurred during racing or training, were included in the study. Each horse was first given a clinical evaluation, and then assessed with both 2D-US and SE within two weeks from the onset of injury.

Before performing the ultrasonographic examination, the hair was cut on the palmar metacarpus, which was then washed thoroughly with water. A coupling gel was finally applied in order to improve probe contact and obtain a better resolution.

Horses were not sedated because they were all sufficiently calm.

The study was approved by the ethics committee (Organismo Preposto al Benessere Animale) of the University of Sassari (protocol code no. 128528, approved on 15 November 2019).

### 2.2. Ultrasound Technique and Devices

Ultrasonographic examinations were performed by one veterinarian (VS) using an ultrasound unit (My Lab Alpha, Esaote, Florence, Italy) equipped with the SE software “ElaXto” (Esaote, Florence, Italy) and a linear 3–13-MHz transducer.

Horses were evaluated in a square stance, under weight-bearing conditions, and forelimbs were assessed from the carpometacarpal joint to the distal sesamoid bones, dividing the palmar aspect of the metacarpus into seven levels (1–7) for transverse planes, and into three levels (1–3) for longitudinal planes. This system differentiates the metacarpal regions according to specific anatomic features and was essential for a better comparison of the images of the various horses involved in the study [15].

The examinations were performed from proximal to distal, first in transverse then in longitudinal planes, at each level of the metacarpus. The probe was positioned in order to obtain lateral structures on the left and medial structures on the right side of the screen, in transverse orientation, and distal parts on the left and proximal parts on the right, in longitudinal orientation.

In order to obtain correct 2D-US images and to avoid anisotropy artefacts, the transducer was held perpendicularly to the surface analyzed [16]. A standoff pad was also used for the 2D-US examinations.

In the trials, power (100%), frequency (intermediate), number and position of foci and depth (~4 cm) were kept stable.

### 2.3. Elastographic Evaluation

Strain elastography was performed by the same trained veterinarian (VS) by applying a gentle manual rhythmic pressure with the linear transducer. As recommended by the manufacturer, the manual compression was minimal, i.e., a vibration with a movement equal or less than 1 mm [5]. A standoff pad was not used in the SE examination.

A color-coded map was selected, with a range of red (softer tissues) through yellow/green (intermediate stiffness) to blue (stiffer tissues) colors.

A wide Fov was set and kept constant (depth of ~4 cm) to obtain as detailed a chromatic scale as possible [5]. In these experimental trials, the Fov included SDFT, deep digital flexor tendon, accessory ligament of deep digital flexor tendon, suspensory ligament, and palmar border of the cannon bone.

A 2D-US image was placed simultaneously beside the elastogram to ensure that the image was kept constant and the probe was not moved laterally, and to assess the movement of tissue during compression–release cycles (1 mm maximum). A minimal precompression was applied.

**Figure 1:**
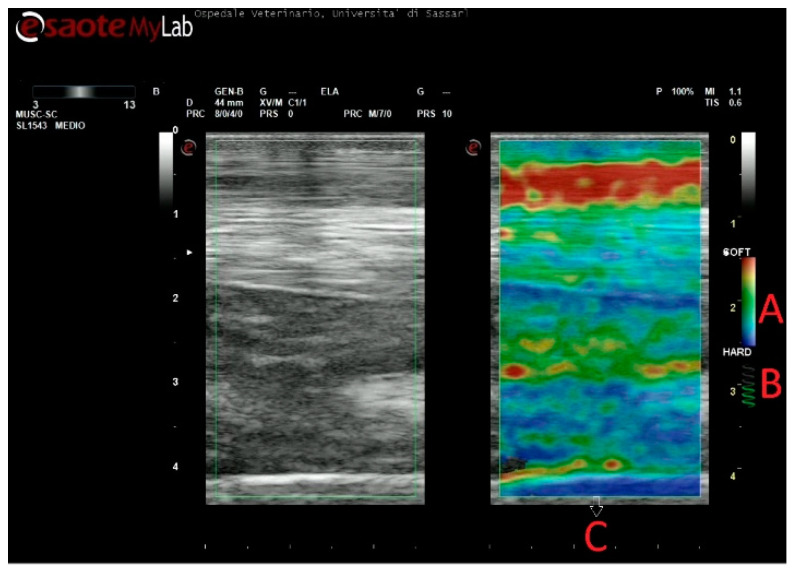
Example of strain elastography. (**A**) Color range selected, (**B**) Indicator for correct compression, (**C**) Field of view.

The accuracy of the elastograms obtained was evaluated during the examination by checking a visual indicator. As the correct compression was applied, the indicator color changed from grey to green, and a videotape was recorded for each level of the metacarpus (Figure 1).

Only images in long-axis view were selected for the following analysis.

### 2.4. Image Analysis

Twenty images, one of each horse, were randomly selected from the most representative videotapes. Elastograms were considered selectable if they enclosed an SDFT lesion and if the indicator for correct compression was green. The same images were then analyzed on the same ultrasound unit by two different veterinarians (VS and AC), who were blinded to each other’s results, in order to test interobserver reproducibility. Image analysis was repeated by one vet (VS) two weeks apart, in order to test intraobserver repeatability.

Image analysis consisted of a measurement of the total area of the SDFT on the longitudinal scan selected and of the area of each predominant color within the tendon (red, green and blue). All measurements were performed three times, using the manual tools available on the ultrasound unit, and the mean values were used for further statistical evaluation. Areas were expressed in cm^2^.

The same images were then objectively evaluated with ImageJ (Version 1.44) [17].

The evaluation was made on a laptop (Ideapad 310-15IKB, Lenovo, Hong Kong, China). Original images (in PNG format), including all the metacarpal structures, were imported into the laptop from the ultrasound unit and cropped along the SDFT perimeter with a basic image editing program; no other manipulation was performed.

Cropped elastograms showing the injured SDFTs were imported in PNG format and analyzed with ImageJ. In each image, the tendon total area and areas of the main colors (red, green and blue) were measured with the “Threshold Color” plugin.

**Figure 2:**
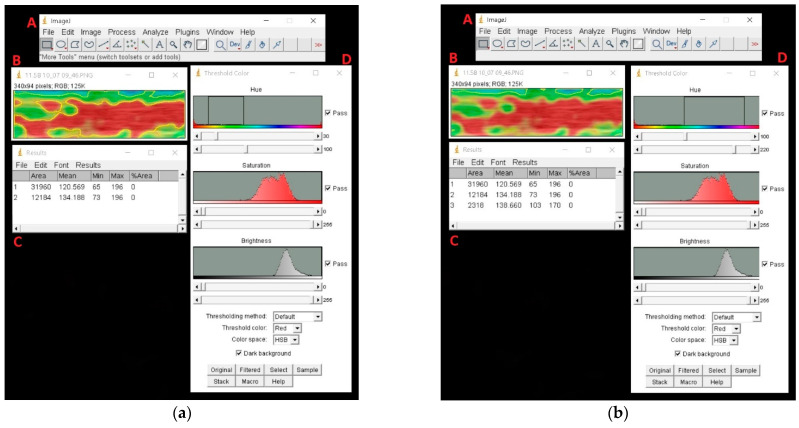
Example of analysis on ImageJ: (**a**) Selection of green area; (**b**) Selection of blue area. The selection of colors is mainly regulated with the Hue bar, and areas (expressed in pixels) of the selected part are measured. (A = ImageJ main tool bar, B = superficial digital flexor tendon elastogram image imported, C = Measurement results, D = “Threshold color” plugin).

For the selection of colors, the Hue bar was the main tool used, with total hues ranging from 0 (red hues) to 255 (red hues). For the selection of green and blue, a range of hues from 30 to 100 and from 100 to 220 was chosen, respectively. On the other hand, the red area was obtained by subtracting green and blue areas from the total areas. Areas were expressed in numbers of pixels (Figure 2).

All the results were recorded in an ad hoc database, and the percentage areas of each color was calculated from the mean values derived from the three consecutive measurements.

### 2.5. Statistical Analysis

The statistical analysis was performed using *R* (Version 3.6.1) [18].

The observed data were triplets $\left(x_{red},x_{green},x_{blue}\right)$ such that $x_{red}+x_{green}+x_{blue}=100\%$. For example, $x_{red}$ represented the ratio between the red area and the total area of the image, expressed as a percentage. A similar interpretation was given to $x_{green}$ and $x_{blue}$. Such data structures describe the parts of a whole and are known in the statistical literature as *compositional data* [19,20]. Mathematically, the observations lie on a 3-part simplex, since $x_{red}$, $x_{green}$ and $x_{blue}$ are constrained to add up to 100%. This constraint violates the assumptions of many standard statistical methods for data analysis. The observations should therefore be projected from the simplex to a 2D real space for further processing. An appropriate transformation is the *isometric log ratio transform* (ILR) [21]. For each observation $\left(x_{red},x_{green},x_{blue}\right)$, the ILR transformation returns a point $\left(y_{1},y_{2}\right)$ in the real plane ${\mathbb{R}}^{2}$ which is suitable for further statistical analysis:

(1) $$\left(y_{1},y_{2}\right)=\mathrm{ILR}\left(x_{red},x_{green},x_{blue}\right).$$

The ILR-transformed values $\left(y_{1},y_{2}\right)$ of the measurements $\left(x_{red},x_{green},x_{blue}\right)$ were used in this work as response variables within the statistical models used for analysis.

A more detailed description of the ILR transform is given in [21]. Practical details regarding the analysis of compositional data in *R* are given in [22], and the associated package compositions in [23]. In this work, the default settings of the package were used to calculate the ILR transformation.

Some of the observations for $x_{blue}$ were exactly zero, which must be substituted before applying ILR. We assumed that the zeros arose for percentages of blue below the detection limit of 1%. The zeros were then replaced using a simple rule of two-thirds of the detection limit. A more detailed description of the replacement strategies in compositional data is given in [24].

Since the response variable was bivariate, two-way repeated measures MANOVA was used to assess intraobserver repeatability, grouped by time of measurement and image, restricted to the observations recorded by the veterinarian (VS). The different images were considered as a random effect, and the resulting mixed model was fitted using the R package *lme4* [25]. The model assumptions were tested using standard statistical tests for multivariate observations: normality was tested using Mardia’s skewness and kurtosis tests (implemented in the R package *MVN* [26]), and homogeneity of the covariance matrices was tested using Box’s M-test (implemented in the R package *heplots* [27]). The models with and without effects for the time of measurement were fitted via maximum likelihood, and the likelihood ratio test was used to assess the difference between them. The adjusted intraclass correlation coefficient (ICC, [28]) was also calculated, with confidence intervals estimated via bootstrapping.

In order to further strengthen the results, intraobserver repeatability was assessed using a one-sample Hotelling’s $T^{2}$-test (implemented in the R package *ICSNP* [29]), applied on the difference between the two measurements of VS. The Mahalanobis distance calculated for the $T^{2}$-test was used as a measure of effect size. Bland–Altman plots [30] were also used to visually assess the agreement between the two measurements, separately for each dimension, after the ILR transformation. In order to provide an intuitive interpretation of the results, the boxplots of the differences between the two measurements were also plotted for each of the three color channels.

Similarly, two-way mixed-effects MANOVA was used to evaluate interobserver repeatability, with grouping factors represented by the veterinarian and the image. The image was considered as a random effect, whereas the veterinarian was assumed to be a fixed effect. Model assumptions were tested using the same procedures used for the study on intraobserver repeatability. The likelihood ratio test was used to assess whether the difference between the measurements of the two veterinarians was statistically significant, and the ICC was calculated with bootstrapped confidence intervals. Bland–Altman plots were constructed from an average of the two measurements of VS, compared to the results obtained by AC. Boxplots for the three color channels were used to provide an intuitive interpretation of the results.

Finally, the difference between the methodologies (manual and ImageJ) was tested using two-way mixed-effects MANOVA, fitted on the entire dataset using maximum likelihood, assuming methodology as the fixed effect and image as the random effect. The underlying model assumptions were again tested using the same procedures described for the two previous studies. The difference between the two methodologies was tested using the likelihood ratio test, which tested the significance of the model coefficients associated with the manual method and ImageJ. In addition, Bland–Altman plots were used to visually assess the agreement between the two methodologies; the plots were constructed from an average of the three measurements from the manual method, compared to the single measurement obtained from ImageJ. As before, boxplots of the three color channels were also used to visually assess the difference between the two methodologies. The threshold for significance of the results of each statistical test was assumed to be α= 0.05. The criteria of [31] were used to evaluate the ICC: poor, (0–0.50); moderate, (0.5–0.75); good, (0.75–0.9); excellent, (0.9–1).

## 3. Results

**Figure 3:**
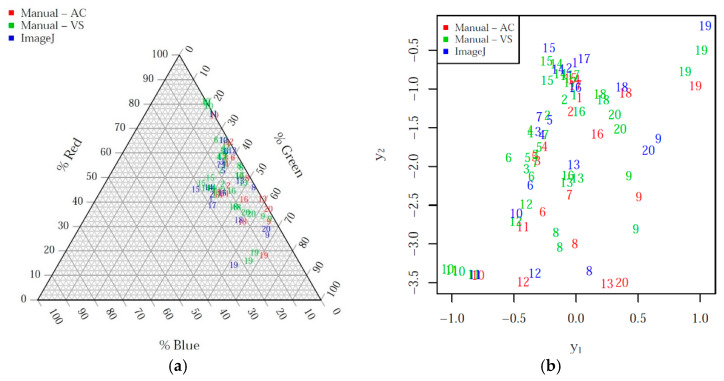
Scatterplot of the measurements before (**a**) and after (**b**) the isometric log ratio (ILR) transformation, labeled by image and colored by observer and method.

Figure 3 shows the scatterplots of the measurements before and after the ILR transformation. Table 1 reports the summary statistics of the ILR-transformed data, and Table 2 the corresponding tests for validation of the MANOVA assumptions. The Mardia and Box’s *M*-test showed no statistical evidence of violations of any of the model assumptions. The mean and confidence interval for the mean of each of the two dimensions $\left(y_{1},y_{2}\right)$ suggested that the two measurements of VS were in agreement, whereas, as expected, more substantial differences were observed in the measurements taken by the different observers (VS and AC) and in the methods (manual and ImageJ).

**Figure 4:**
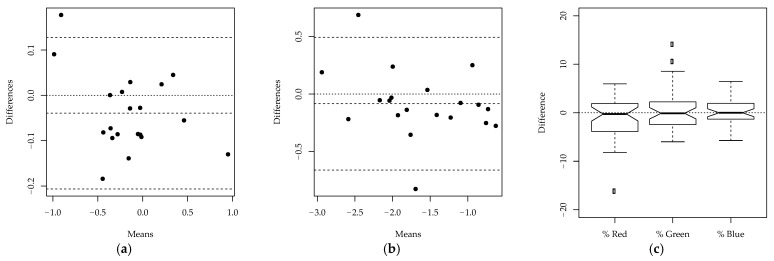
Intraobserver reproducibility study: (**a**) Bland-Altman plot—First dimension of isometric log ratio transformed data; (**b**) Bland-Altman plot—Second dimension of isometric log ratio transformed data; (**c**) Boxplots of the differences between the first and second measurement by VS for each color channel.

Intraobserver repeatability, interobserver reproducibility and the difference between the manual method and ImageJ were more precisely quantified by the likelihood ratio tests reported in Table 3. The two measurements by VS on the same images did not significantly differ (*p*-value 0.157), demonstrating the intraobserver repeatability of the methodology. This was confirmed by Hotelling’s $T^{2}$-test on the differences between the measurements on individual images (*p*-value 0.097). The ICC further demonstrated that most of the variability was explained by the differences between the images (adjusted ICC 0.949, with the lower bound of the 95% confidence interval exceeding 0.9), showing excellent intraobserver repeatability. The agreement between the two measurements is also confirmed by Figure 4, which shows the Bland–Altman plots on each of the two dimensions of the ILR-transformed data, and the boxplot of the difference between the measurements for each color channel. In the Bland–Altman plots, the observations do not significantly deviate from the horizontal line at zero. In addition, in the boxplots, the differences for each color channel appear to have a symmetric distribution around zero, further demonstrating the intraobserver repeatability of the methodology.

**Figure 5:**
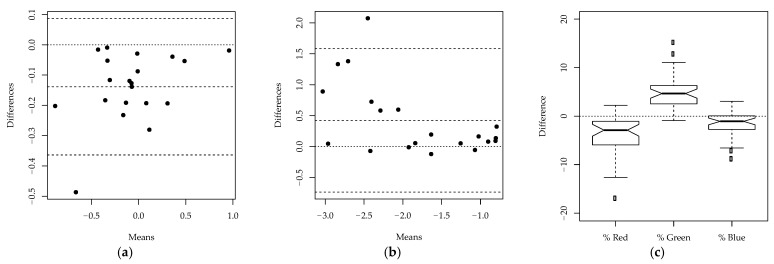
Interobserver reproducibility study: (**a**) Bland-Altman plot—First dimension of isometric log ratio transformed data; (**b**) Bland-Altman plot—Second dimension of isometric log ratio transformed data; (**c**) Boxplots of the differences between the measurements of AC and VS for each color channel.

Similar statistical tests were carried out to assess interobserver reproducibility. From the likelihood ratio test shown in Table 4, it appeared that the measurements taken by the veterinarians, AC and VS, were significantly different (*p*-value < 0.001). This is confirmed by Figure 5, as the observations in the Bland–Altman plots are not scattered around the horizontal line at zero, and the boxplots of the differences for the three color channels are not centered around zero. A significant difference was observed between the two veterinarians in the assessment of the percentages of the colors red and green. Despite the significant difference between the two observers, the adjusted ICC (0.855, with the lower bound of the 95% confidence interval exceeding 0.75) reported in Table 4 still suggests that there is good agreement between the two veterinarians’ measurements, since most of the variability is due to the differences between the images. The images refer to lesions with different gravity levels and are therefore highly heterogeneous. The variability between the measurements on different images is thus much larger than the variability of measurements on the same image, which leads to large values of the ICC.

**Figure 6:**
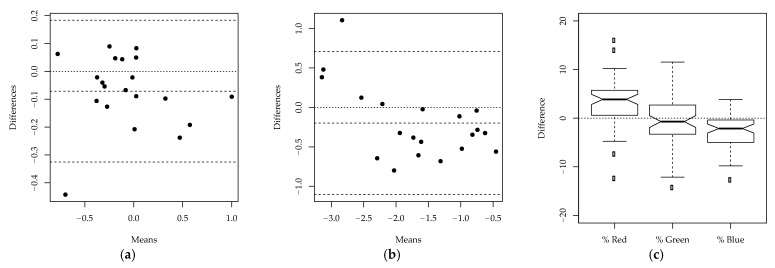
Agreement between manual method and ImageJ: (**a**) Bland-Altman plot—First dimension of ILR-transformed data; (**b**) Bland-Altman plot—Second dimension of ILR-transformed data; (**c**) Boxplots of the differences between the measurements obtained using manual method and ImageJ, for each color channel.

Finally, the difference between the measurements obtained using the manual method and ImageJ was assessed. The likelihood ratio test in Table 5 suggests that there is a statistically significant difference between the measurements obtained using the two methodologies (*p*-value < 0.001). Again, this is confirmed by the Bland–Altman plots and boxplots reported in Figure 6: the observations in the Bland–Altman plots are not randomly distributed around zero, and the boxplots of the differences for the three color channels show that the measurements of the colors red and blue significantly deviate from zero. The adjusted ICC (0.849, with the lower bound of the 95% confidence interval exceeding 0.75) in Table 5 demonstrates that, despite the significant differences between the two methodologies, there is good agreement between the measurements. Similarly to the study on interobserver reproducibility, the ICC is large because most of the variability is due to the differences between images.

## 4. Discussion

The manual measurements of colors in the elastograms of acutely injured SDFTs in horses showed excellent intraobserver repeatability (ICC 0.949) and good interobserver reproducibility (ICC 0.855). Good agreement was also obtained between the manual method and the ImageJ methodologies of the color area measurements (ICC 0.849).

The manual measurement of elastograms was thus shown to be a repeatable and reproducible method which could be considered as interchangeable with the objective measurement obtained with the software image analysis.

Our results confirmed previous findings showing that SE image analysis had an almost perfect interobserver agreement when imaging injured tendons [11]. Moreover, our results showed a higher level of agreement compared to a previous study conducted on healthy tendons, in which the qualitative evaluation had moderate interobserver agreement and good intraobserver agreement [6]. Only one study reported the variability associated both with acquiring and analyzing images, which was reported as low [6]. To the best of our knowledge, in equine medicine, no study has reported the standardization of the manual analysis of SE-derived elastograms using image analysis software.

Strain elastography is a qualitative USE technique and is performed by applying a gentle manual rhythmic compression on tissues. It does not produce numerical elasticity parameters, but a relative representation of the elasticity of tissues within the selected window. It is highly operator-dependent, requiring well-trained sonographers. In addition, accurate acquisitions involve careful preparation [5,7,32,33,34].

Healthy equine SDFTs normally appear in elastograms as predominantly blue structures, with a mostly green peritendinous tissue, and surrounded by soft tissue structures, which appear as red [6]. When a lesion occurs, during the acute inflammatory phase, the injured area of the tendons becomes softer (red), due to hemorrhage, edema and fibrin clot organization [35]. As the granulation tissue forms, tendons become more stable, and the color changes to yellow/green, representing intermediate stiffness [36,37]. During the remodeling phase, tendons become progressively harder, returning to their original mechanical properties (mainly blue) [38].

In order to obtain accurate elastograms of SDFTs in horses, animals should be evaluated under weight-bearing conditions, because there are usually fewer artefacts in the elastographic evaluation compared to a non-weight-bearing position [6,11]. Minimal precompression should be used in order to prevent precompression artefacts. In fact, when excessive precompression is exerted, elastograms are not representative of the real mechanical characteristics of tissues [5]. The visual indicator for correct compression does not consider the precompression degree and thus could appear green despite an insufficiently accurate evaluation. In our study, elastographic examinations of the SDFT were relatively easy to perform, due to the superficial location of the examined structure, thus obtaining a qualitative representation of the mechanical properties of pathologic tendons. We only analyzed longitudinal images due to problems encountered in obtaining elastograms in the short-axis view, especially in distal levels of the metacarpus. Images of transverse planes showed more artefacts than longitudinal ones, especially on medial and lateral parts, due to the convex margins of the tendons. Both in human medicine, for the evaluation of Achilles tendons, and in veterinary medicine, for the evaluation of SDFTs in horses and calcaneal and patellar tendons in dogs, longitudinal images are preferred because they are considered to be of better quality, while transverse images show more artefacts and lower reproducibility [12,39,40,41].

**Figure 7:**
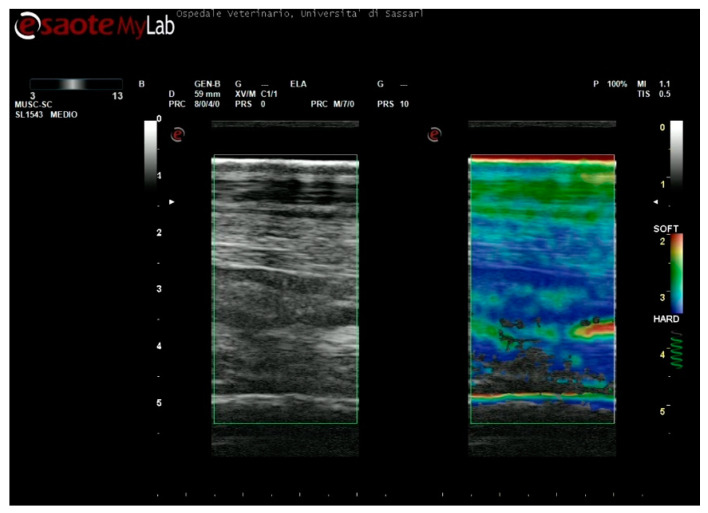
At the bottom of the field of view (Fov), an artifact is present as a lack of signal, without any colors. This could be explained by the use of the pad, as the deepest structures, where the artifact appears, are probably more difficult to reach, compared with images obtained without a pad. Secondly, the inclusion of a standoff pad within Fov can alter the color pattern of strain elastography-derived elastograms, because of its relativity; the standoff pad can represent the softest part within elastographic window, and as a consequence, the superficial digital flexor tendon lesions can be misinterpreted or underestimated, no longer appearing red but yellow/green.

The SE images were obtained without a standoff pad, which can be problematic for a correct evaluation of the elasticity pattern. The lack of accurate contact between the probe and standoff pad can produce reverberation artifacts, which are visible as parallel red areas in the elastogram, which can be confused with areas of softness [6,42]. In addition, the inclusion of the pad in the Fov can alter the color pattern of SE-derived elastograms, because of its well-known relativity. The standoff pad can represent the softest part within the elastographic window, and, as a consequence, SDFT lesions can be misinterpreted or underestimated, no longer appearing as red but yellow/green—in other words, with intermediate stiffness. Finally, the use of the pad can alter the elasticity estimation since the deepest structures, which we decided to include (suspensory ligament and cannon bone), are probably more difficult to reach. This can subsequently lead to a lack of signal, without any color (Figure 7).

In our trials, it was not necessary to sedate the horses; however, the use of a sedative is recommended if needed. The influence of sedation on USE results is still controversial, with some authors stating that the results are not compromised [6,12] and others declaring that images can be potentially influenced by sedation [43].

For the SE image analysis, categorical color-grading is normally the most common system used. It is a qualitative method which was first used in human medicine for the evaluation of Achilles tendons [13,14] and subsequently adapted for equine SDFTs [6,11]. This simple categorical assessment facilitates a semi-quantitative analysis of data, with high interobserver agreement and applicability in clinical practice [11,12].

However, due the subjective nature of color-grading, we used ImageJ for a more objective analysis, which we compared with a manual assessment using an ultrasound unit. As recommended [44,45], the evaluation consisted in calculating the percentage of the three main colors present in the elastograms: red (softest structures), green (intermediate stiffness) and blue (stiffest areas).

The software analysis was performed with the “Threshold Color” plugin, because it was considered easier to interpret clinically and more reproducible than qualitative or quantitative methods, which measure the mean echo-intensity for each color considered [44,46,47].

A significant difference was initially observed between the two veterinarians’ (VS and AC) measurements and the measurements taken using the two methodologies However, the subsequent analyses established that most of the variability was due to differences between images; thus, in reality, good ICCs were obtained.

Differences between images could be explained considering different sizes, distributions and shapes of the colored areas which characterize injured tendon elastograms. For example, measuring an area with a regular and larger shape is simpler than measuring an area with an irregular and smaller shape. This can be explained clinically by the heterogeneity of the SDFT lesions included in the sample. As a consequence, some elastograms were very different than others, in terms of the proportion, morphology and margins of colors within Fov.

Moreover, the differences in tracing could also be a product of the differences in the visual perception of colors, caused by the wide range of hues in the elastograms. Different shapes and irregular distributions of colors could also make the visual perception problematic.

The study has several limitations.

Firstly, a small sample size was recruited, with a relative heterogeneity regarding the region and gravity of disease. However, patients were homogenous concerning breed, activity and the time of diagnosis from the injury onset.

Secondly, two-dimensional ultrasonography and SE are both considered highly operator-dependent methods and, especially for SE, several precautions had to be taken, as previously explained. For this reason, all the evaluations were performed by the same trained veterinarian (VS). In addition, strain elastography is a type of USE that shows a relative categorization of tissue elasticity. Other methods, such as SWE, which provide objective numerical parameters, could be more reliable, and the data obtained may be easier to analyze. Moreover, USE is still not commonly used in veterinary medicine; thus, more studies and comparisons are needed in order to standardize the method and subsequent analysis. However, this is still difficult due to the high variability of procedures and analysis techniques present in the literature.

No histopathologic assessment was performed, since all cases consisted of racehorses undergoing rehabilitation in order to return to competition.

Finally, image analysis using ImageJ was a long and complex procedure and several parameters needed to be considered. Despite the easy automatic selection and measurement of colored areas by the software, ranges of hue, saturation and brightness (Figure 1) had to be previously selected and tested by the operator, since a minimal change in range selection of each parameter can influence the results. To prove this, an evaluation was performed, considering different configurations of hue, brightness and saturation, showing significant differences between image categorizations. For this reason, a standardization of the selection of ranges is needed in order to make the assessment as accurate as possible, which should correspond as much as possible to the manual assessment. Furthermore, the low use of ImageJ and especially of the “Threshold Color” plugin in veterinary medicine provided few models for comparison.

## 5. Conclusions

This study demonstrates that the manual measurements of colors within elastograms of pathologic tendons showed good and excellent inter- and intraobserver reliability, respectively.

In addition, good agreement was also obtained between the manual and ImageJ analyses, for the evaluation of injured SDFTs in horses. We believe that this demonstrates that manual measurement is a feasible and accurate method and that it could be considered as valid as the objective software assessment. As an accurate interpretation and measurement of lesions is fundamental, it could thus be used to evaluate tendon healing.

Future studies are recommended to better evaluate the potential of SE for musculo-skeletal structures and different imaging analysis techniques in horses.

## Figures and Tables

**Table 1:** Summary (mean and 95% Student’s t confidence interval for the mean) of the isometric log ratio (ILR)-transformed data.

Method	Observer	$y_{1}(1\mathrm{st}\;\mathrm{Dimension}\;\mathrm{of}\;\mathrm{ILR})$		$y_{2}(2\mathrm{nd}\;\mathrm{Dimension}\;\mathrm{of}\;\mathrm{ILR})$	
		Mean	95% C.I.	Mean	95% C.I.
Manual	VS (Measurement 1)	−0.174	(−0.371, 0.023)	−1.812	(−2.195, −1.429)
	VS (Measurement 2)	−0.134	(−0.346, 0.078)	−1.728	(−2.146, −1.309)
	AC	−0.015	(−0.198, 0.168)	−2.062	(−2.519, −1.606)
ImageJ	VS	−0.037	(−0.238, 0.164)	−1.582	(−2.044, −1.120)

**Table 2:** Statistical tests for validation of the mixed-effects model assumptions.

Method	Observer	Mardia’s *p*-Values		Box’s M *p*-Value
		Skewness	Kurtosis	(∼Grouping)
Manual	VS (Measurement 1)	0.314	0.559	0.940 (∼measurement)
	VS (Measurement 2)	0.246	0.538	
	AC	0.615	0.546	0.429 (∼observer)
ImageJ	VS	0.299	0.711	0.957 (∼method)

**Table 3:** Likelihood ratio test, intraclass correlation coefficient and Hotelling’s $T^{2}$-test for the intraobserver repeatability study. ICC, Intraclass Correlation Coefficient; C.I., Confidence Interval.

Likelihood Ratio Test		Adjusted ICC		$\mathrm{Hotelling}’s\;T^{2}-\mathrm{Test}$	
$\chi^{2}\mathrm{Score}$	*p*-Value	Estimate	95% C.I.	Mahalanobis Distance	*p*-Value
3.698	0.157	0.949	(0.905, 0.972)	2.711	0.094

**Table 4:** Likelihood ratio test and intraclass correlation coefficient for the interobserver reproducibility study. ICC, Intraclass Correlation Coefficient; C.I., Confidence Interval.

Likelihood Ratio Test		Adjusted ICC	
$\chi^{2}\;\mathrm{Score}$	*p*-Value	Estimate	95% C.I.
18.250	<0.001	0.855	(0.753, 0.907)

**Table 5:** Likelihood ratio test and intraclass correlation coefficient for the study on the differences between the manual method and ImageJ. ICC, Intraclass Correlation Coefficient; C.I., Confidence Interval.

Likelihood Ratio Test		Adjusted ICC	
$\chi^{2}\mathrm{Score}$	*p*-Value	Estimate	95% C.I.
16.076	<0.001	0.849	(0.751, 0.899)

## Data Availability

Data are available on request to the authors.
